# Silkworm Thorn Stem Extract Targets RSK2 and Suppresses Solar UV-Induced Cyclooxygenase-2 Expression

**DOI:** 10.3390/ijms161025096

**Published:** 2015-10-22

**Authors:** Jong-Eun Kim, Ki Won Lee

**Affiliations:** 1 WCU Biomodulation Major, Department of Agricultural Biotechnology and Center for Food and Bioconvergence, Seoul National University, Seoul 151-921, Korea; E-Mail: idonlike@gmail.com; 2 The Hormel Institute, University of Minnesota, Austin, MN 55912, USA; 3 Advanced Institutes of Convergence Technology, Seoul National University, Suwon 443-270, Korea

**Keywords:** silkworm thorn, cyclooxygenase-2, ultraviolet, RSK2

## Abstract

Excessive exposure to solar UV (sUV) is associated with numerous human skin disorders, such as carcinogenesis, skin photoaging and skin inflammation. Silkworm Thorn (*Cudraniatricuspidata*, SW) is a plant belonging to the Moraceae family and widely present throughout Korea, China, and Japan. Most parts of the tree (including the fruit, leaf, stem, root, and bark) is consumable as a functional food or tea. In this study, we found that SW extract (SWE) inhibited the elevated expression of sUV-induced cyclooxygenase (COX)-2 levels in both HaCaT and JB6 cells. Levels of nuclear factor-κB and activator protein-1, two crucial transcription factors involved in COX-2 expression, were elevated by sUV treatment. Treatment with SWE abolished this activation. SWE also inhibited sUV-induced histone H3 phosphorylation. However, sUV-induced phosphorylation of Akt, c-Jun N-terminal kinase and p38 kinase remained unchanged in the presence of SWE. SWE inhibited RSK2 activity, and pull-down assays using SWE-Sepharose beads revealed that SWE binds directly with RSK2 in an ATP-competitive manner. These results suggest a potential for SWE to be developed as a cosmeceutical material and functional food constituent for the promotion of skin health.

## 1. Introduction

Ultraviolet (UV) is a major etiological factor involved in skin photoaging and carcinogenesis [1]. Exposure of the skin to UV induces damage of DNA, protein, and lipids, which can in turn lead to skin inflammation, a process that precedes photoaging and carcinogenesis [2]. UV is classified into the subtypes UVA, UVB, and UVC, with UVA having the longest wavelength at 320–400 nm, and UVB ranging from 290 to 320 nm [3]. Most UVC is absorbed by the Earth's ozone layer and does not impact human health [4,5]. As a result, Solar UV (sUV) measured at sea level consists of approximately 95% UVA and 5% UVB [6]. Areas of thinning in the ozone layer allow more UV to reach the ground, increasing the risk of adverse effects [7]. Therefore, methods to inhibit sUV-induced skin inflammation may help to counteract these adverse effects.

Silkworm Thorn (*Cudraniatricuspidata*, SW) is a plant belonging to the Moraceae family, and is widely distributed throughout Korea, China, and Japan. Almost all parts of the tree are edible [8], and it has been used as a traditional medicinal ingredient in Korea and China for the treatment of pneumonia, influenza, hyperpiesia and phthisis [9]. The bark of SW has been traditionally valued as a treatment for inflammatory diseases such as neuritis, bruising and rheumatism [10]. Several studies have shown that SW elicits potentially beneficial effects on human health. For example, the fruit of SW has been observed to suppress Dermatophagoides farina-induced development of atopic dermatitis in NC/Nga mice [11], while its leaf material has been shown to inhibit lipase activity *in vitro* and lipid absorption in rats [12]. In addition, the stem bark of SW induces apoptosis in human HL-60 leukemia cells [10] and inhibits LPS-induced production of prostaglandin E2 and nitric oxide in RAW 264.7 macrophages [13]. However, the effects of SW on sUV-induced skin inflammation have not previously been investigated.

Cyclooxygenase (COX)-2 plays a crucial role in sUV-induced skin inflammation [14]. COX is the rate-limiting enzyme in the production of prostaglandins (PGs), which are inflammatory lipids [15]. There are two major isoforms of COX, COX-1 and COX-2 [16]. COX-1 is normally constitutively expressed, but COX-2 is inducibly activated by stimuli, including growth factors, bacterial endotoxins, cytokines, hormones, and sUV [15]. PGs which are produced by COX-2 are involved in many pathophysiological processes such as inflammation, angiogenesis pain, vasodilation, fever, wound repair, and increases in vascular permeability [14]. Chronic exposure to sUV irradiation promotes COX-2 expression and COX-2-mediated sUV-induced skin inflammation [17]. In contrast, COX-2 inhibition prevents skin inflammation and aging [18,19], representing a potential strategy for preventing skin inflammation and skin aging. Here, we investigated the inhibitory effects of SW extract (SWE) on sUV-induced COX-2 expression in skin cells.

## 2. Results

### 2.1. SWE Inhibits sUV-Induced COX-2 Expression through Transcriptional Suppression

**Figure 1:**
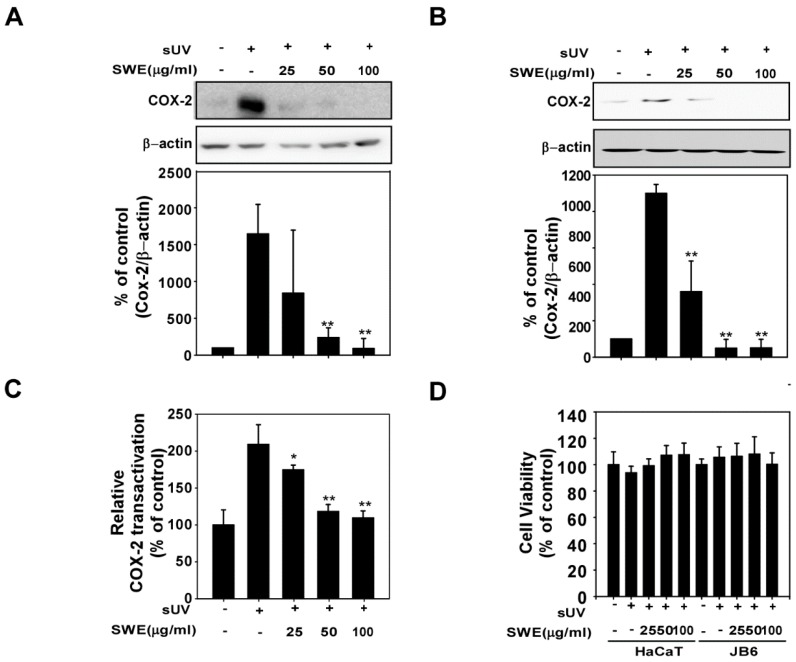
Effects of Silkworm Thorn stem extract (SWE) on solar ultraviolet (sUV)-induced cyclooxygenase (COX)-2 expression in JB6 and HaCaT cells. (**A**,**B**) SWE inhibits sUV-induced COX-2 expression in (**A**) JB6 and (**B**) HaCaT cells. The cells were treated with SWE at the concentrations indicated (0, 25, 50, or 100 µg/mL) for 1 h before exposure to 90 kJ/m^2^ sUV and harvested 4 h later. COX-2 and β-actin (control) expression levels were assessed by Western blotting. COX-2 expression was quantified using the Image J software program; (**C**) SWE suppresses sUV-induced *cox-2* promoter transactivation activity. JB6 cells were stably transfected with COX-2 luciferase reporter plasmids and treated with SWE at the indicated concentrations (0, 25, 50, or 100 µg/mL) for 1 h before exposure to 90 kJ/m^2^ sUV and prepared 6 h later. Relative activity was determined using a luciferase assay as described in the Materials and Methods; (**D**) Cell viability was measured using MTT assay described in the Materials and Methods. The data are presented as the mean ± S.D. Data are shown as mean  ±  SD and asterisks indicate a significant inhibition by SWE compared with the group treated with sUV alone (* *p* < 0.05 and ** *p* < 0.01).

Previous studies have reported that COX-2 is closely associated with skin inflammation. We therefore sought to evaluate the effect of SWE on sUV-induced COX-2 expression in JB6 and HaCaT cells. SWE treatment dose-dependently downregulated sUV-induced COX-2 expression in JB6 (Figure 1A) and HaCaT cells (Figure 1B) without affecting cell viability (Figure 1D). To measure whether COX-2 inhibition by SWE through transcriptional regulation, we showed the effects of SWE on sUV-induced *cox-2* promoter activity. Luciferase assays revealed that exposure to sUV induced *cox-2* promoter activity significantly and this induction was suppressed by SWE treatment (Figure 1C).

### 2.2. SWE Inhibits sUV Induced NF-κB/AP-1 Transactivation and Histone H3 Phosphorylation

**Figure 2:**
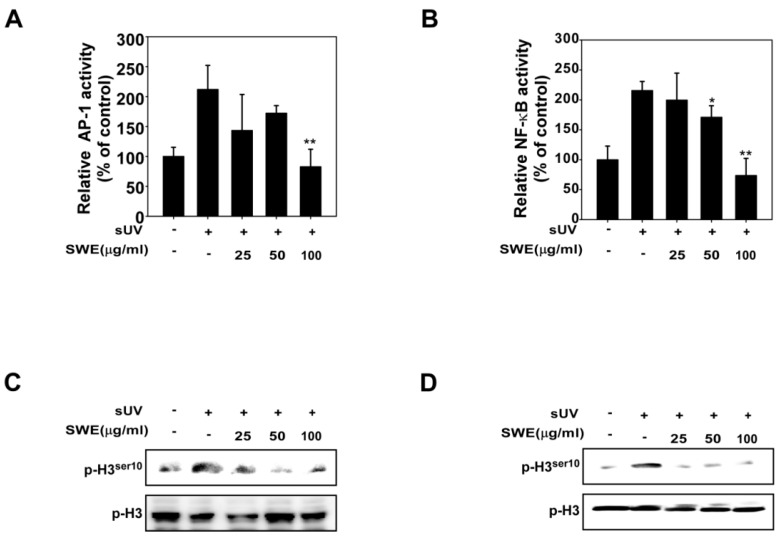
Effects of SWE on sUV-induced AP-1/NF- B transactivation and histone H3 Phosphorylation. (**A**,**B**) SWE suppresses sUV-induced AP-1 and NF- B transactivation. JB6 P+ cells, which were stably transfected with either AP-1 or NF- B luciferase reporter plasmids, were treated with SWE at the concentrations indicated (0, 25, 50, or 100 µg/mL) for 1 h before exposure to 90 kJ/m^2^ sUV and prepared 6 h later. Luciferase assay as described in the Materials and Methods; (**C**,**D**) SWE suppresses sUV-induced histone H3 phosphorylation. (**C**) JB6 and (**D**) HaCaT cells were treated with SWE at the concentrations indicated (0, 25, 50, or 100 µg/mL) for 1 h before exposure to 90 kJ/m^2^ sUV and harvested 1 h later using an acid preparation as described in the Materials and Methods. Western blot analysis was conducted using specific antibodies as indicated. Data are presented as mean values ± SD (* indicates *p* < 0.05, ** indicates *p* < 0.01).

Exposure to sUV stimulates the activation of NF-κB and AP-1, which are crucial transcription factors involved in COX-2 expression and skin inflammation. We assessed the effect of SWE on NF-κB and AP-1 transactivation using JB6 cells stably transfected with NF-κB or AP-1 luciferase reporter plasmids. Consistent with the results for COX-2 expression, SWE inhibited sUV-induced transactivation of AP-1 (Figure 2A) and NF-κB (Figure 2B), which may relate to the anti-inflammatory properties of SWE. A previous study has shown that UV-induced COX-2 expression occurs via AP-1, mediated by histone H3 phosphorylation [20]. We investigated the effects of SWE on sUV-induced histone H3 phosphorylation, and found that SWE inhibits sUV-induced histone H3 phosphorylation in JB6 (Figure 2C) and HaCaT (Figure 2D) cells.

### 2.3. SWE Does Not Inhibit sUV-Induced MAPK or Akt Signaling Activity

**Figure 3:**
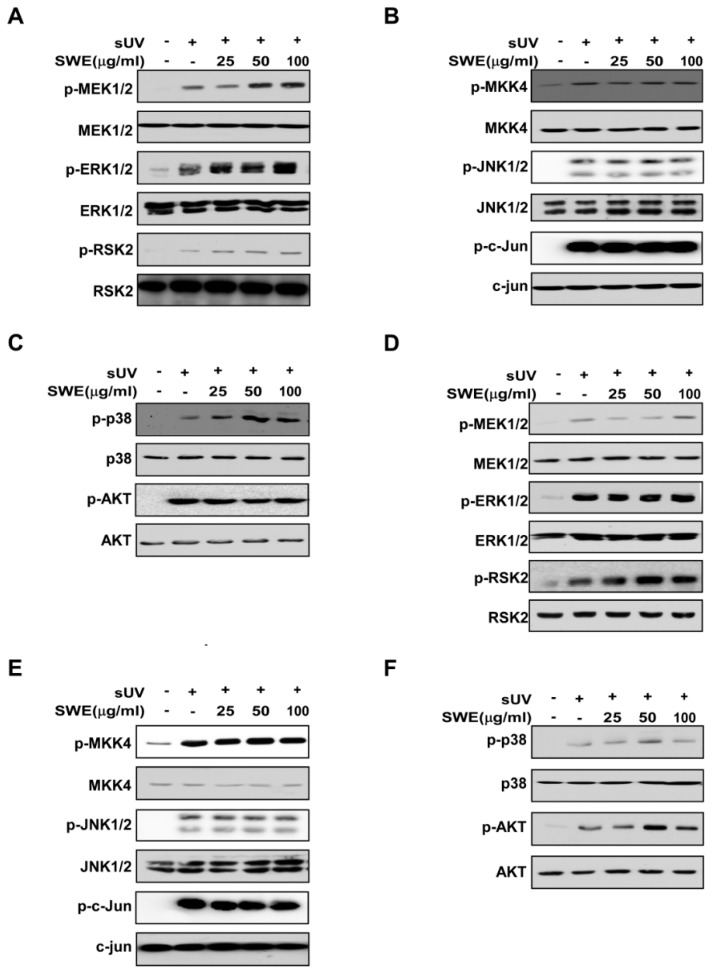
Effects of SWE on sUV-induced signaling in JB6 and HaCaT cells. JB6 (**A**–**C**) and HaCaT (**D**–**F**) cells were treated with SWE at the indicated concentrations (0, 25, 50, or 100 μg/mL) for 1 h before exposure to 90 kJ/m^2^ sUV and harvested 30 min later. Western blot analysis was performed as described in the Materials and Methods using indicated specific antibodies.

Transcriptional activation of sUV-induced COX-2 expression is known to be regulated by the MAPK and Akt signaling pathways [21]. However, we found that SWE did not inhibit UV-induced ERK1/2, p38 MAPK, JNK1/2 or Akt expression in JB6 or HaCaT cells (Figure 3). These findings suggest that factors downstream of the MAPK and Akt pathways, or other pathways are responsible for the effects of SWE on sUV-induced COX-2 expression in JB6 and HaCaT cells.

### 2.4. SWE Inhibits RSK2 Activity though Direct Binding

**Figure 4:**
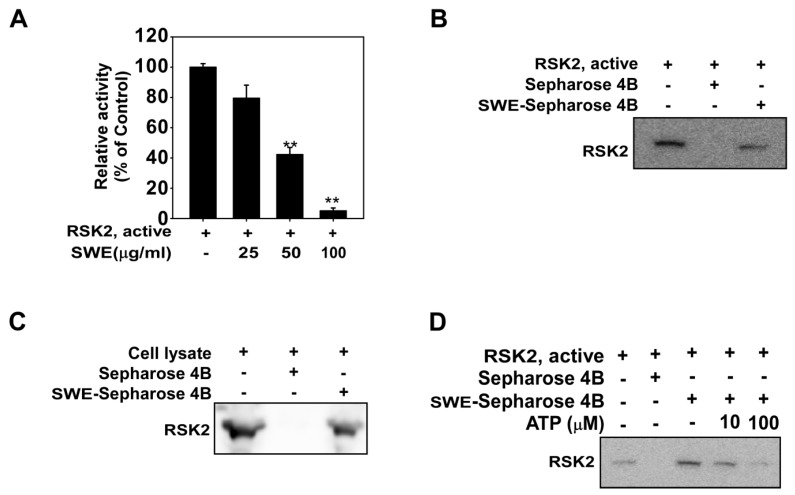
Effects of SWE on ribosomal S6 kinase 2 (RSK2) activity with direct binding. (**A**) SWE inhibits RSK2 activity. Active RSK2 protein was incubated with SWE at the indicated concentrations at 30 °C for 15 min. Kinase activity was measured as described in the Materials and Methods. The asterisks (**) indicate a significant difference (*p* < 0.01) compared to the untreated kinase control; (**B**) Binding of SWE to RSK2. RSK2–SWE binding was confirmed by immunoblotting using an antibody against SWE: Lane 1 (input control), RSK2, protein standard; Lane 2 (control), Sepharose 4B was used for pull-down assay as described in the Materials and Methods; Lane 3, RSK2 was pulled down using SWE–Sepharose 4B beads; (**C**) SWE binds to sUV-activated RSK2. SWE binding in sUV-exposed JB6 cells was confirmed by Western blot analysis using an antibody against RSK2: Lane 1 (input control), JB6 cell lysate; Lane 2 (control), JB6 cell lysate were precipitated with Sepharose 4B beads; Lane 3, JB6 cell lysate were precipitated with SWE–Sepharose 4B; (**D**) SWE binds to RSK2 in an ATP-competitive manner: Lane 1 (input control), active RSK2; Lane 2 (control), active RSK2 precipitated with Sepharose 4B; Lane 3, active RSK2 precipitated with SWE–Sepharose 4B; Lane 4, active RSK2 precipitated with SWE–Sepharose 4B in the presence of ATP 10 µM; Lane 5, active RSK2 precipitated with SWE–Sepharose 4B in the presence of ATP 100 µM.

Although SWE did not inhibit RSK2 phosphorylation (Figure 3), its functional target, the histone H3 phosphorylation which is a substrate of RSK2 was downregulated by SWE (Figure 2C,D). Based on these findings, we postulated that SWE is inhibiting RSK2 activity. Using an *in vitro* kinase assay, we showed that RSK2 activity was reduced in a concentration-dependent manner by SWE (Figure 4A). To identify how SWE modulates RSK2 activity, we investigated whether SWE binds directly to RSK2. Pull-down assay results revealed that SWE physically binds to the active RSK2 protein (Figure 4B, Lane 3), but not to unconjugated Sepharose 4B beads (Figure 4B). We also observed binding of SWE to RSK2 in JB6 cells (Figure 4C). Next, to examine the mode of SWE binding to RSK2, we conducted ATP competitive-binding assays. ATP was observed to compete with SWE for RSK2 binding (Figure 4D), suggesting that SWE binds to or otherwise affects the RSK2 ATP-binding pocket.

## 3. Discussion

The ribosomal S6 kinase (RSK) is an AGC kinase of the RSK family and a prominent serine/threonine kinase [22]. This family consists of four human isoforms RSK1–4 [22]. In particular, RSK2 plays a crucial role in skin inflammation and carcinogenesis [23]. RSK2 is comprised of two kinase domains designated as the N-terminal kinase domain (NTKD) and the C-terminal kinase domain (CTKD), an N-terminal domain (NTD), linker region (LR) and C-terminal domain (CTD). It is activated downstream of the MEK/ERKs cascade by cellular stress factors such as sUV [24]. RSK2 is known to play pivotal roles in the activation of transcription factors involved in inflammation, cell proliferation transformation and cancer development. Previous studies have shown that the inhibition of RSK2 prevents skin cancer and inflammation [25]. In this study, we observed that the inhibitory effects of SWE on sUV-induced COX-2 expression appear to occur through direct inhibition of RSK2.

SWE has been shown to elicit various bioactive effects that are potentially beneficial for human health including antibiotic, anti-inflammatory, antioxidant and anticancer effects [10,26,27,28,29,30,31,32]. But, the mechanisms responsible for these findings have not been elucidated. In this study, we observed that at least some effects of SWE on sUV-induced skin inflammation appear to occur via inhibition of COX-2 expression. We also postulate that RSK2 is a direct target of SWE, as treatment inhibited RSK2 activity and phosphorylation of histone H3. Histone H3 phosphorylation provides a mechanistic link to sUV-induced COX-2 expression [20]. Previous studies showed that SWE contains various bioactive constituents including glutinol, taraxerone, quercetin, isorhamnetin, orobol and kaempferol [33,34]. Of particular note, kaempferol suppresses RSK2 activity and was observed to elicit inhibitory effects on sUV-induced COX-2 expression in our previous studies [22,35,36].

COX-2 is a critical player in the processes of skin inflammation and skin carcinogenesis, and has also been implicated in photoaging. Elevated COX-2 levels have been detected in photoaged skin [19], with aged human skin containing more COX-2 after UV exposure than younger skin [18]. COX-2 signaling has therefore become a therapeutic target of interest for skin health. Strategies for the inhibition of COX-2 include the inhibition of direct enzyme activity and protein expression. Although a number of COX-2 inhibitors have been developed, side effects remain a major issue, with reports of renal and hepatic insufficiency and gastrointestinal erosions, which are thought to arise due to off-target COX-1 inhibition [37]. However, the specific inhibition of COX-2 is thought to be possible without serious side effects [14,38]. SWE has been shown to inhibit sUV-induced COX-2 expression and has been used as a dietary ingredient and oriental medicine for a significant period of time [11,30]. If the effects of SWE can be elucidated in clinical studies, it may eventually be accepted for use as a commercial cosmeceutical material and functional food for skin health.

In conclusion, skin inflammation is a crucial process in skin carcinogenesis and photoaging [7,17,20,36]. SWE is a natural botanical extract which has been used as a food source and oriental medicine [11,30]. In this study, we showed that SWE suppresses sUV-induced COX-2 expression, which is a major biomarker of skin inflammation and found that it directly targets RSK2.

## 4. Experimental Section

### 4.1. Materials

SWE was obtained from the Plant Extract Bank (PEB) at the Korea Research Institute of Bioscience and Biotechnology (distribution number: 026-021). Stems of SW were collected from fields in Korea and cut into small pieces and dried naturally under shade. SW was extracted using methanol (MeOH), and stored at −20 °C until use. A stock solution (100 mg/mL) was prepared in dimethyl sulfoxide (DMSO). 3-[4,5-dimethylatiazol-2-yl]-2,5 diphenyltetrazolium bromide (MTT) powder was purchased from USB Co. (Cleveland, OH, USA). The antibodies against The antibodies against phosphorylated ERKs (Thr202/Tyr204) and total ERK, total MEK1/2, total MAPKK4 were from Santa Cruz Biotechnology (Santa Cruz, CA, USA) and phosphorylated MAPKK3/6 (Ser189/207), total MAPKK3/6, phosphorylated MAPKK4 (Ser257/Thr261) phosphorylated MEK1/2 (Ser217/221) and total p90^RSK^ were purchased from Cell Signaling Technology (Beverly, MA, USA). Anti-β-actin was purchased from Sigma–Aldrich (St. Louis, MO, USA). RSK2 assay kits were obtained from Merck Millipore (Lake Placid, NY, USA). Cyanogen bromide–Sepharose 4B, [γ-^32^P] adenosine triphosphate (ATP) and the chemiluminescence detection kit were obtained from GE Biosciences (Piscataway, NJ, USA), and the protein assay kit was acquired from Bio-Rad Laboratories (Hercules, CA, USA).

### 4.2. Cell Culture and sUV Radiation

JB6 P+ mouse skin epidermal cells (JB6 Cells, 5% FBS-MEM) and HaCaT cells (10% FBS-DMEM were cultured at 37 °C with 5% CO_2_ in growth medium supplemented with antibiotics. Cells were maintained by subculturing at 70% to 85% confluence and growth media was changed every 3 days. sUV was generated by UVA-340 lamps purchased from QLab Corporation (Cleveland, OH, USA). The UVA-340 lamps provide an optimal simulation of sunlight within the critical short wavelength region from 365 nm down to the solar cutoff of 295 nm with a peak emission of 340 nm. The percentage of UVA and UVB produced by these lamps was measured by a UV meter at 94.5% and 5.5%, respectively. The standard dose of sUV used in this study was 90 kJ/m^2^.

### 4.3. Western Blot Analysis

Cultured cells were prepared and washed twice. After centrifugation at 12,000 rpm for 5 min, cells were resuspended with 200 μL RIPA buffer [50 mM tris-HCl at pH 8.0, 1% NP-40, 0.5% deoxycholate, 150 mM NaCl, 0.1% SDS, 1 mM Na_3_VO_4_, 1 mM dithiothreitol, 1 mM phenylmethylsulfonyl fluoride (PMSF)] for protein extraction. For the isolation of histone proteins, cells were resuspended in 180 μL of 1× PBS and 20 μL of 2 N HCl (final concentration of 0.2 N HCl) and incubated on ice for 30 min. The lysate was centrifuged at 15,000 rpm for 10 min at 4 °C. The supernatant fraction was then mixed with 100 μL 50% trichloroacetic acid and incubated on ice for 30 min. The precipitated proteins were washed twice with acetone and dissolved in H_2_O. Proteins were resolved by SDS-PAGE and transferred onto nitrocellulose membranes (Merck Millipore), which were blocked with skim milk and hybridized with specific 1st antibodies. The protein bands were visualized using ECL solution after hybridization with a horseradish peroxidase–conjugated 2nd antibody.

### 4.4. Luciferase Assays for cox-2 Promoter Activity and Induced Nuclear Factor-κB (NF-κB) and Activator Protein (AP)-1 Transcriptional Activity

Confluent monolayers of JB6 cells stably transfected with NF-κB, AP-1 or *cox-2* luciferase reporter plasmids were seeded in 96-well plate. Plates were incubated at 37 °C in a 5% CO_2_ incubator. At 80%–90% confluence, cells were transferred to 0.1% FBS/MEM for 24 h. The cells were then treated with various concentrations of SWE for 1 h and then exposed to 90 kJ/m^2^ sUV and harvested after 6 h to assess *cox-2*, NF-κB or AP-1 activity. After treatment, cells were disrupted with 100 µL lysis buffer (0.1 M potassium phosphate buffer (pH 7.8), 1% Triton X-100, 2 mM EDTA, 1 mM DTT), and luciferase activity was measured using a luminometer (Luminoskan Ascent, Thermo Electron, Helsinki, Finland).

### 4.5. Cell Viability

Cell viability was measured by MTT assay. HaCaT and JB6 cells were cultured in 96 well plates at a density of 4 × 10^5^ cells/well and were incubated. After reaching 80% cell confluence, the HaCaT cells and JB6 cell were starved in serum free media for 24 h. The cells treated with SWE as indicated concentration were incubated for 22 h at 37 °C. After incubation, MTT solution was treated for 2 h. DMSO is used for dissolving formazan crystals. A microplate reader (Molecular Devices, Sunnyvale, CA, USA) was used to measure at 570 nm.

### 4.6. In Vitro RSK2 Kinase Assay

RSK2 kinase assays were performed followed by following the manufacturer’s instructions (Merck Millipore). Briefly, SWE and active RSK2 were incubated at 30 °C for 15 min in resaction buffer (20 mM MOPS (pH 7.2), 5 mM EGTA, 25 mM β-glycerol phosphate, 1 mM sodium orthovanadate and 1 mM DTT). Two μL of RSK2 Substrate Peptide 2 (KKRNRTLTK) was added to each mixture, then incubated at 30 °C for 15 min with [γ-^32^P] ATP solution in a magnesium acetate–ATP cocktail buffer. The reaction mixture was then transferred onto p81 paper. Using 0.75% phosphoric acid, the p81 paper was washed three times for 5 min. The radio-labeled phosphate was measured using a scintillation counter.

### 4.7. SWE Pull-down Assay

Recombinant human RSK2 (0.2 μg) and JB6 cell lysates were incubated with SWE-Sepharose 4B beads or Sepharose 4B-only as a control in reaction buffer [50 mM Tris (pH 7.5), 5 mM EDTA, 150 mM NaCl, 0.01% NP40, 1 mM DTT, 0.02 mM PMSF, 2 μg/mL bovine serum albumin, 1× protease inhibitor mixture]. After incubation with gentle shaking overnight at 4 °C, the beads were washed five times with buffer [50 mM Tris (pH 7.5), 5 mM EDTA, 150 mM NaCl, 1 mM DTT, 0.01% NP-40, 0.02 mM PMSF], and proteins bound to the beads were analyzed by Western blotting.

### 4.8. Statistical Analysis

Data are expressed as the mean values ± standard deviation (SD). Student’s *t*-test was used to evaluate statistical significance between groups. Results were considered statistically significant at *p* < 0.05.
